# Altered irisin/BDNF axis parallels excessive daytime sleepiness in obstructive sleep apnea patients

**DOI:** 10.1186/s12931-019-1033-y

**Published:** 2019-04-05

**Authors:** Csaba E. More, Csaba Papp, Szilvia Harsanyi, Rudolf Gesztelyi, Angela Mikaczo, Gabor Tajti, Laszlo Kardos, Ildiko Seres, Hajnalka Lorincz, Krisztina Csapo, Judit Zsuga

**Affiliations:** 1 Department of Psychiatry, Faculty of Medicine, University of Debrecen, Nagyerdei krt. 98, Debrecen, 4032 Hungary; 2 Department of Health Systems Management and Quality Management for Health Care, Faculty of Public Health, University of Debrecen, Nagyerdei krt. 98, Debrecen, 4032 Hungary; 3 Department of Pharmacology and Pharmacotherapy, Faculty of Medicine, University of Debrecen, Nagyerdei krt. 98, Debrecen, 4032 Hungary; 4 Department of Pulmonology, Faculty of Medicine, University of Debrecen, Nagyerdei krt. 98, Debrecen, 4032 Hungary; 5 Institute of Clinical Pharmacology, Infectious Diseases and Allergology, Kenezy Gyula Teaching County Hospital and Outpatient Clinic, Bartok Bela ut 2-26, Debrecen, 4031 Hungary; 6 Department of Internal Medicine, Faculty of Medicine, University of Debrecen, Nagyerdei krt. 98, Debrecen, 4032 Hungary; 7 Department of Neurology, Faculty of Medicine, University of Debrecen, Moricz Zsigmond krt. 22, Debrecen, 4032 Hungary

**Keywords:** Irisin, BDNF, ESS, Polysomnography, Circadian rhythm

## Abstract

**Study objectives:**

Obstructive sleep apnea hypopnea syndrome (OSAHS) is a sleep-related breathing disorder, characterized by excessive daytime sleepiness (EDS), paralleled by intermittent collapse of the upper airway. EDS may be the symptom of OSAHS per se but may also be due to the alteration of central circadian regulation. Irisin is a putative myokine and has been shown to induce BDNF expression in several sites of the brain. BDNF is a key factor regulating photic entrainment and consequent circadian alignment and adaptation to the environment. Therefore, we hypothesized that EDS accompanying OSAHS is reflected by alteration of irisin/BDNF axis.

**Methods:**

Case history, routine laboratory parameters, serum irisin and BDNF levels, polysomnographic measures and Epworth Sleepiness Scale questionnaire (ESS) were performed in a cohort of OSAHS patients (*n* = 69). Simple and then multiple linear regression was used to evaluate data.

**Results:**

We found that EDS reflected by the ESS is associated with higher serum irisin and BDNF levels; β: 1.53; CI: 0.35, 6.15; *p* = 0.012 and β: 0.014; CI: 0.0.005, 0.023; *p* = 0.02, respectively. Furthermore, influence of irisin and BDNF was significant even if the model accounted for their interaction (*p* = 0.006 for the terms serum irisin, serum BDNF and their interaction). Furthermore, a concentration-dependent effect of both serum irisin and BDNF was evidenced with respect to their influence on the ESS.

**Conclusions:**

These results suggest that the irisin-BDNF axis influences subjective daytime sleepiness in OSAS patients reflected by the ESS. These results further imply the possible disruption of the circadian regulation in OSAHS. Future interventional studies are needed to confirm this observation.

## Introduction

Obstructive sleep apnea hypopnea syndrome (OSAHS) is gaining increased attention given its profound societal and long-term, health related consequences. Reports regarding its prevalence reflect great variability with some reflecting a prevalence of 3–7% and 2–5% in men and women, respectively [1], while others indicating prevalence as high as 36.5% [2]. OSAHS is a sleep-related breathing disorder characterized by excessive daytime sleepiness paralleled by intermittent collapse of upper airway leading to impaired gas exchange and consequent hypoxemia, events often terminated by arousal [3, 4]. Airway pathology triggers sympathetic hyperactivation, inflammation and oxidative stress paving the way along the continuum of cardio-metabolic diseases, by contributing to endothelial dysfunction, insulin resistance, and subsequently results in arterial hypertension, diabetes mellitus, stroke, myocardial infarction, heart failure and sudden death [1, 4]. Furthermore, an independent association between OSAHS and cardiovascular as well as all-cause mortality has been shown in clinical studies [5, 6].Excessive daytime sleepiness per se, a canonical symptom of OSAHS, leads to more frequent motor vehicle accidents, diminished work capacity and productivity, and is causative in approximately 9.1% of work-related accidents [2].

Adaptation to the daily fluctuation of light and dark periods is the most fundamental challenge for an organism, that basically determines its physiology and behavior. It is orchestrated by the hierarchically organized circadian system, the central master clock and peripheral slave clocks present in most tissues. The master clock resides in the suprachiasmatic nucleus (SCN). It has an intrinsic pacemaker activity with its free-running intrinsic circadian period being longer than the 24 h long environmental photoperiod [7]. Hence, continuous adjustment (entrainment) is mandatory. The SCN is synchronized to geophysical time mainly through photic signals, and it conveys the rhythm of the environment to distinct brain areas and peripheral organs by neuronal and endocrine signals e.g. by changing sympathetic outflow [8], core body temperature, melatonin levels, and sleep each adding to the circadian regularity of the organism [9, 10]. Insufficient entrainment of the master clock causes metabolic syndrome [11], obesity and diabetes [12], cardiovascular disease [13], depression [14], cancer [15], and dementia [16, 17]. The SCN is most readily entrained by light, in a brain-derived neurotrophic factor (BDNF) dependent manner, rendering BDNF the gatekeeper, regulating light’s ability to shift the phase of the master clock. BDNF is a well circumscribed neurotrophin implicated in maintaining cerebral neuroplasticity [18], by inducing long-term potentiation and consequent increase of the strength of synaptic connections [19]. Building on recent preclinical and clinical observations that BDNF expression may be induced by irisin, a putative contraction-regulated myokine, we have theorized that irisin, either by crossing the blood-brain-barrier, or by its direct expression in the hypothalamus is able to increase BDNF levels in the retinohypothalamic synapse, and hence may modulate BDNF’s effect on photic entrainment [20]. Research denoting that peripheral signals, e.g. exercise and fasting, both known to be upstream regulators of irisin expression, are only able to shift the master clock if light input is simultaneously present further corroborate this presumption [21].

Sleep regulation is well conceptualized by the two-process model of sleep, that posits the continuous interaction of oscillating homeostatic processes (growing and declining sleep debt experienced during wakefulness, and sleep, respectively) and the circadian pacemaker process, governed by the master clock. As a result, the phase, amplitude and periodicity of oscillations are entrained in a way that sleep is properly aligned with the 24-h day-night cycle [22]. Optimal timing of sleep with regards to the circadian phase is an important regulatory signal aligning the central circadian and peripheral tissue specific rhythms [22, 23]. Homeostatic and circadian pacemaker processes interact albeit are regulated separately.

Circadian misalignment e.g. altered sleep phase, with respect to the circadian environment, and/or altered amplitude of underlying circadian processes leads to diminished sleep quality and excessive sleepiness, and impaired cognitive performance [24, 25]. Shift in the circadian phase per se was shown to have a significant effect on subjective sleepiness, an effect that was more pronounced in younger (average age 24.5 ± 3.54 years) vs. older (average age 64.0 ± 5.98 years) adults using the forced desynchrony paradigm [26], hence excessive daytime sleepiness may be indicative of altered circadian regulation.

Excessive daytime sleepiness is a well characterized phenomenon of OSAHS, in fact, it was found to be a significant and independent predictor of incident cardiovascular events and all-cause mortality in a historical cohort of 10,149 participants with a follow-up time of 68 months [27]. Moreover, the phenomena of residual sleepiness, e.g. excessive daytime sleepiness in OSAHS patients treated effectively with continuous positive airway pressure (CPAP) therapy, implies that the underlying pathology of excessive daytime sleepiness is different from that of OSAHS per se*.* Conversely, presence oxidative stress and systemic inflammation [1] and a significant association with all-cause and cardiovascular morbidity and mortality have been reported as a consequence of residual sleepiness in OSAHS patients [28, 29]. The need for distinct therapeutic approaches for managing sleep-related breathing disorders and excessive daytime sleepiness have also been articulated [1].

Alteration of the circadian rhythm has been implicated in OSAHS. Blunted daily variability of the expression of Per1, one of the regulatory genes of the circadian master clock has been reported along with excessive daytime sleepiness, mood disturbances and increased incidence of cardiovascular disease [30, 31]. Conversely, several studies have provided evidence for the adverse effect of OSAHS on the circadian secretion of melatonin, considered as one of the main neural output signals of SCN [24] and a predictable marker of circadian phase [22]. Synthetized in the pineal gland, this serotonin-derived hormone has a short half-life with no depot, rendering circulating melatonin levels highly responsive to light. Melatonin synthesis is entrained to the ambient light-dark cycle [32], its levels are low during the day, and begin to rise in evening (in dim light settings of < 50 Lux) peaking between midnight and 2 AM [33, 34]. Both endogenous and exogenous melatonin has been shown to induce sleepiness, via their influence on the circadian component of sleep regulation [35]. Alteration in the function of the master circadian clock implicated by phase shift or change in the amplitude of melatonin secretion has been reported in OSAHS patients [34, 36, 37]. Improper functioning of the master circadian clock was further suggested by Lemmer and colleagues, who reported loss of nocturnal peak parallel to the emergence of a prominent peak in melatonin secretion around 4 AM. Interestingly, 8 weeks of CPAP therapy failed to alter melatonin levels, blood pressure and heart rate, while significantly improved deep sleep, slow wave sleep, rapid eye movement sleep, arousal index, apnea–hypopnea index, oxygen desaturation index, and the 24 h plasma norepinephrine levels [37]. Based on these considerations, we propose that that disruption of circadian rhythm is not a consequence of the mechanical obstruction of the upper airways, rather it is a co-evolving pathology further increasing the disease burden in OSAHS. This notion is further underscored by the known phenomenon of residual sleepiness [1].

In the present study we tested the hypothesis that impaired circadian rhythm reflected by subjective daytime sleepiness is influenced by the irisin/BDNF axis, given that irisin may be able to influence the expression level of BDNF in the SCN, at the site of photic entrainment, of a cohort of polysomnography verified OSAHS patients.

## Methods

### Study design and protocol

The present study was designed in line with the STROBE statement for cross-sectional studies [38] and was approved by the Ethical Committee of the University of Debrecen (DEOEC RKEB/IKEB 3715–2012). Informed consent was obtained from each participant. The investigation conforms to the principles outlined in the Declaration of Helsinki.

The current study is based on the analysis of a cohort of patients who attended the accredited Sleep Medicine Center of the Department of Neurology (University of Debrecen) between October 1, 2012 and April 30, 2013 and was diagnosed to have OSAHS. Accreditation was made by the Hungarian Society for Sleep Medicine in compliance with European guidelines for the accreditation of Sleep Medicine Centres [39]. Every patient meeting the diagnostic criteria for OSAHS according to the relevant Hungarian [40] and concordant international guidelines [41] were invited to participate, given they met the inclusion criteria and failed to meet any of the exclusion criteria. Inclusion criteria were age between 18 and 80 years and diagnosis of OSAHS at the time of inclusion. The clinical diagnosis of OSAHS was made if the number of obstructive events (apneas, hypopneas + respiratory event related arousals) on polysomnography was greater than 5 events/hour, and the patient reported excessive daytime sleepiness and/or at least two of the following: repeated nighttime awakening, unrefreshing sleep, decreased concentration and impaired memory, fatigue, repeated gasping or choking while asleep. Sleep related events were scored according to standard operational procedures [42] and the manual of the American Academy of Sleep Medicine [43]. Exclusion criteria included inability to provide informed consent, pregnancy, kidney disease, bronchial asthma or chronic obstructive pulmonary disease (COPD), inflammatory diseases of the face or oral cavity, any systemic autoimmune disease.

Overall, 70 patients were recruited, data from one patient was excluded from the analysis due to a highly significant outlier (serum irisin > 15 ng/mL). Patients were diagnosed with OSAHS at the time of their recruitment, thus received no therapy at inclusion for this syndrome. Each patient underwent a comprehensive sleep evaluation, neurological examination, polysomnography, and laboratory examination, demographic, anthropometric, anamnestic data were also acquired. Common carotid artery intima-media thickness (IMT) was analyzed offline as described previously [44]. In addition, the Pittsburg Sleep Quality Questionnaire, the Epworth Sleepiness Scale and the Beck depression scale were filled out. Patients received therapy for their co-morbidities as clinically warranted.

### Polysomnography

In-laboratory full-night polysomnography was performed according to the European standard operational procedures (Fisher 2011) (Philips Alice IV and V, Respiromed, Hungary. Patients were directed to take their medication as usual on the night of their sleep study. Patients were mounted with 6 EEG electrodes (F3-M2, C3-M2, C4-M1, O2-M1), 2 EOG electrodes, 1 submental EMG, ECG (one channel), pulsoxymeter, body position sensor, nasal pressure/thermal flow sensor, snore sensor. Proper, artefact-free functioning of the recording devices, trouble-shooting was provided by the continuous oversight of nurses certified in clinical electrophysiology. Every sleep study was reviewed and interpreted by a qualified sleep physician. Severity of OSAHS was quantified by the apnea-hypopnea index (AHI), and the patient sample was dichotomized for the purpose of descriptive statistics using the cutoff for AHI at < 30/h (mild to moderate severity vs severe OSAHS) [41]. The following parameters were included in the statistical analysis: AHI, oxygen desaturation index (1/h), arousal index (1/ h), central apnea index (1/ h), obstructive apnea index (1/ h),, hypopnea index (1/ h), oxygen saturation below 90% (%).

### Blood samples

After an overnight fast blood, samples were drawn in the morning of the examination. Routine laboratory investigations were performed according to the standard clinical practice of the Department of Laboratory Medicine (University of Debrecen), making use of the locally used reference values. Serum or plasma samples were used for determining measures descriptive of carbohydrate homeostasis (glucose, insulin, hemoglobin A1c (HgA1c)), lipid homeostasis (total cholesterol, triglyceride, LDL-cholesterol, HDL-cholesterol, Lp(a), apoA1, apoB), kidney function (urea, creatinine), liver function (GOT, GPT, γGT), status of skeletal muscles (CK, LDH) and systemic inflammation (C-reactive protein (CRP)). CRP was dichotomized as high vs. normal with the cutoff being 4.6 mg/L and 5.2 mg/L for female and male patients, respectively. Serum samples used to determine irisin and BDNF were frozen within 60 min and stored at − 80 °C until further analysis.

### Determination of serum irisin and BDNF

BDNF is synthetized in a pre-pro form and may be released into the circulation as pro-BDNF or mature BDNF (referred to as BDNF throughout the text) and it can also cross the blood-brain barrier. The most important stimulus for the expression and release of BDNF is excitatory synaptic activity that by releasing glutamate into the synapse leads to ligand- or voltage-gated neuronal influx of Na^+^ and Ca^2+^ subsequently activating neuronal transcription factors and inducing BDNF gene transcription. BDNF is then transported to presynaptic axon terminals and dendrites and is released upon glutamate receptor activation [45]. Serum BDNF levels were measured in accordance with the manufacturer’s protocol (Sigma-Aldrich, MO, USA). In short, standards and samples were administered into anti-BDNF monoclonal antibody coated 96-well microplates in 100X dilution and were incubated overnight at 4 °C. Plates were then washed 4 times and 100 μl of biotinylated anti-human BDNF antibody was added. The samples were incubated for 1 h with continuous gentle shaking. Afterwards, the wells were washed again and 100 μl HRP-Streptavidin solution was added to the wells for a 45-min long incubation period at room temperature with gentle shaking. After subsequent washing, 100 μl TMB One-Step Substrate Reagent was added and incubation was undertaken for 30 min to induce the reaction leading to colored substrate. The reaction was stopped by adding the manufacturer-supplied stop solution. The absorbance was measured at 450 nm with an automatic microplate reader. The detection limit for BDNF in our experiment was lower than 80 pg/ml.

Irisin, a highly conservative 12 kDa polypeptide [46], is formed by proteolysis of the transmembrane protein fibronectin type III domain containing 5 (FNDC5). Expression of FNDC5 is controlled by peroxisome proliferator-activated receptor-gamma coactivator protein-1α (PGC1α), a transcriptional co-activator regulating oxidative metabolism in brown adipose tissue [47]. Expression of both, PGC1α and FNDC5 may be induced by physical activity and fasting [48, 49]. After proteolytic cleavage of FNDC5, irisin enters the systemic circulation where it can readily cross the blood-brain barrier. Albeit most abundant in skeletal muscle, FNDC5/irisin expression is significant in the adipose tissue [46], tongue, rectum, and brain while lower levels of irisin were detected in the kidney, liver and lung [50]. Additional to the effect currently scrutinized, e.g. inducing BDNF synthesis in the central nervous system, irisin’s most profound effect is increasing oxygen consumption and thermogenesis of fat cells [47] by inducing white adipose tissue browning in various regions [51] via upregulating thermogenic genes such as uncoupling protein 1 (UCP-1) and PGC1α. Serum irisin levels were assayed as written in the manufacturer-supplied instruction manual. We applied a commercially available ELISA kit (Phoenix Pharmaceuticals, Burlingame, CA, USA). Briefly, 50 μl of 2X diluted standard or sample, 25 μl primary antibody and 25 μl biotinylated peptide was added to each well for a 2-h long incubation period at room temperature. Afterwards, wells were washed four times and 100 μl SA-HRP solution was added for 1 h at room temperature. After the necessary washing step, 100 μl of substrate solution was added to each well followed by incubation for 1 h, after which the reaction was stopped with 100 μl/well of 2 M HCl. Absorbance was measured subsequently at 450 nm. According to the manufacturer’s description, the standard curve of irisin was linear from 1.34 to 29.0 ng/ml, and the detection limit was 1.34 ng/ml. A standard curve (concentration against optical density) of irisin as well as BDNF were obtained with each plate. The curves showed linear relationship within the range of our measurements.

### Questionnaires

Several questionnaires were completed by the study participants in a supervised administration setting. To assess habitual daytime sleepiness the Epworth Sleepiness Scale (ESS) [52], one of the most widely used questionnaires, was administered. The ESS is developed to provide a subjective measure of daytime sleepiness, and contains 8 scenarios in which susceptible patients are likely to doze off or fall asleep. Patients are asked to assess the probability of dozing for each of these situations using the ordinal scale of 0 to 3 (with 0 and 3 indicating a zero or high chance of dozing), yielding a minimal and maximal total score of 0 and 24, respectively indicative of the severity of excessive sleepiness. The conventional cutoff for excessive sleepiness is drawn at > 10 points. Previously, significant correlations were shown between ESS and the objective measure of sleepiness by the multiple sleep latency test [53]. The Pittsburgh sleep quality index (PSQI) is a self-administered questionnaire that contains 19 questions organized into seven components, each scored on a scale of 0 to 3 reflecting no difficulty to severe difficulty, respectively [54]. The seven components describe subjective sleep quality, sleep latency, sleep duration, habitual sleep efficiency, sleep disturbance, daytime dysfunction and use of sleeping medication. Component scores are summed to yield a global score in the range of 0 to 21 point, where 21 indicates severe difficulties in all areas. Component scores may also be interpreted on their own. Sleep quality is considered to be impaired if the PSQI global score is > 5. This reflects severe difficulties in at least two, or moderate difficulties in more than three areas. Several of these measures may reflect the homeostatic aspects of sleep [55] (e.g. sleep duration, sleep efficiency, sleep disturbance and use of sleeping medications). Additional to the self-rated questions the questionnaire contains 5 questions, to be answered by a roommate. Answers to these questions are not included in the index.

We used the original Beck Depression Inventory comprised of 21 questions descriptive of symptoms and attitudes characteristic of depression. Each question may be evaluated on a scale of 0 to 3 with respect to intensity [56, 57]. Hungarian versions of these questionnaires were provided by the Hungarian Society for Sleep Medicine.

### Statistical analysis

Normality of continuous variables was checked by the Shapiro-Wilk test. In case of Gaussian distribution, Student’s t-test was used for the comparison of two data sets, if not, Mann-Whitney U test was carried out. Frequencies were compared with Pearson’s χ^2^ test.

Demographic, anthropometric, anamnestic, laboratory and polysomnography data were compared based on the severity of the OSAHS, using AHI, with cutoff levels of < 30/h vs ≥ 30/h for mild to moderate severity vs severe OSAHS, respectively).

For linear regression, parameters showing Gaussian distribution were used in their raw forms, whereas those not normally distributed were appropriately transformed to obtain normal distribution. Accordingly, the parameters were transformed as follows: (log) systolic blood pressure, (log) weight, 1/body mass index (1/BMI), (log) abdominal circumference, (log) systolic blood pressure, log (triglyceride), (log) PSQI, (sqrt) arousal index, (sqrt) central apnea index, (sqrt) obstructive apnea index,, (sqrt) hypopnea index, (sqrt) Beck depression score, (log) triglyceride, (log) HDL-cholesterol.

To identify determinants of Epworth Sleepiness Scale Score, serum irisin and BDNF levels, simple linear regression was carried out including traditional confounding factors (age, gender), anthropometric parameters (height, (log) weight, 1/BMI, neck and (log) abdominal circumference), blood pressure ((log) systolic blood pressure, diastolic blood pressure), intima media thickness, polysomnographic parameters, laboratory parameters characteristic of carbohydrate and lipid homeostasis, (log) PSQI and (sqrt) Beck score. Furthermore, information regarding the number of people living in a household, smoking, benzodiazepine use, presence of diabetes mellitus, arterial hypertension, coronary artery disease, cerebrovascular disease, metabolic syndrome (the latter six parameters dichotomized as yes vs. no) were also assessed. Polysomnography parameters yielding significant regressors were combined into a single parameter using principal component analysis. Missing data were omitted. Then, in order to eliminate effects of potential confounders, multiple linear regression modeling was performed including both irisin and BDNF levels, and all significant regressors determined with the simple linear regression as well as age and gender (as a priori variables). Variables were introduced into the initial multiple model simultaneously, then factors not contributing significantly to the model were deleted. The final model contained all variables identified a priori, and (log) PSQI. In addition, the final model was assessed for the interaction of irisin and BDNF. Heteroskedasticity and goodness of fit for the model was assessed by Cook-Weisberg and Ramsey test.

Statistical analysis was performed with Stata 18.0 software (Stata Corporation). Values are given as mean ± SD or median (with the interquartile range: IQR), regression coefficients are presented with their 95% confidence interval (CI).

## Results

### Patients

Data from the 69 patients were analyzed. The average age was 53.81 ± 10.72 years, and 18 patients were female. The baseline characteristics of the cohort of OSAHS patients is summarized in Table 1. Of the 69 patients, 16 patients did not take any medications, 16 patients took statins, 41 patients took antihypertensives (any of the following: ACE inhibitor, ARB, beta receptor blocker, Ca channel antagonist, diuretic, or other), 11 patients took oral antidiabetics, 19 patients received aspirin, 8 patients took benzodiazepines and 10 patients used proton pump inhibitors regularly.

**Table 1:** Baseline characteristics of the study population (*n* = 69 patients)

Parameters	
Age (years)	53.81 ± 10.72
Gender (female/ male)	18/51
Smoker (n/y)	57/12
RR systolic (mmHg)	140 (125–145)
RR diastolic (mmHg)	89.18 ± 9.52
Weight (kg)	94 (86.4–110)
Abdominal circumference	112 (106–123)
Neck circumference	44.10 ± 5.20
Height (m)	171.35 ± 7.63
BMI (kg/m^2^)	31.97 (28.77–37.03)
Irisin (ng/ml)	6.93 ± 8.40
BDNF (ng/ml)	368.80 ± 113.70
Glucose (mmol/L)	5.5 (5.2–6.2)
Cholesterol (mmol/L)	5.18 ± 1.05
Triglyceride (mmol/L)	1.5 (1.1–2.6)
CRP (normal/high)	54/12
Beck depression inventory score	6 (3–10)
Epworth score	10.04 ± 4.63
PSQI	5 (3–8)
AHI (1/h)	37.95 ± 23.97
Oxygen desaturation index (1/h)	25.4 (8.8–63.5)
Arousal index (1/h)	38.4 (20.6–55.4)
Central apnea index (1/h)	1.9 (0.7–4.6)
Obstructive apnea index (1/h)	12.1 (5.1–26.6)
Hypopnea index (1/h)	13.4 (8.8–20.3)

### Comparison of patients regarding OSAHS severity

The two strata of our OSAHS cohort, dichotomized by AHI (cutoff at < 30/h for low to moderate severity) was homogenous with respect to basic demographic data (age and gender distribution) and most parameters (Table 2). However, patients suffering from severe OSAHS had higher prevalence of arterial hypertension, were more obese indicated by significantly higher weight, BMI, larger neck and abdominal circumference. These patients had a less favorable cardiometabolic risk profile indicated by significantly higher IMT, serum glucose and HbA1c, triglyceride Lp(a) and Apo-A1 levels, and lower HDL-cholesterol level. Higher CRP levels and arterial hypertension were also more prevalent in this subset of patients. Naturally polysomnography measures were significantly worse in the subset of patients with severe OSAHS. Furthermore, higher proportion of patients suffered from daytime sleepiness as indicated by the ESS in this patient subset (Table 2). Sleep quality and depression characterized by the PSQI and Beck Depression Inventory showed no significant difference.

**Table 2:** Comparison of the patients with respect to severity of obstructive sleep apnea hypopnea syndrome (OSAHS)

Parameters	Mild to moderate OSA	Severe OSA	*p*
Age (years)	51.52 ± 11.14	55.47 ± 10.23	0.131
Gender (f/m)	11/18	7/33	0.056
Smoker (n/y)	24/5	33/7	0.978
Diabetes (n/y)	26/3	30/10	0.124
CAD (n/y)	25/4	35/5	0.875
CVD (n/y)	27/2	35/5	0.447
Metabolic syndrome (n/y)	3/21	5/34	0.970
RR systolic (mmHg)	135 (120–145)	141.25 (126.25–146.25)	0.300
RR diastolic (mmHg)	87.08 ± 8.23	90.44 ± 10.11	0.174
Arterial hypertension (n/y)	**12/16**	**4/36**	**0.002**
Weight (kg)	**91.2 (76.5–94.7)**	**100.85 (90.25–121.65)**	**< 0.001**
BMI (kg/m^2^)	**30.07 (28.23–32.56)**	**35.51 (30.15–39.07)**	**< 0.001**
Abdominal circumference (cm)	**108 (99–113)**	**118.5 (109–126.5)**	**< 0.001**
Neck circumference (cm)	**40.96 ± 4.03**	**46.35 ± 4.80**	**< 0.001**
Height (m)	169.93 ± 7.57	172.39 ± 7.59	0.189
Irisin (ng/ml)	6.91 ± 0.76	6.94 ± 0.90	0.886
BDNF (ng/ml)	359.12 ± 108.75	375.99 ± 118.12	0.549
IMT (mm)	**0.61 ± 0.09**	**0.67 ± 0.09**	**0.015**
Urea (mmol/L)	5.1 (4.1–6)	5.4 (4.3–6.6)	0.246
Creatinine (μmol/L)	**69 (62–80)**	**80 (74–96)**	**< 0.001**
GOT (U/L)	**18 (17–22)**	**21 (18–25)**	**0.044**
GPT (U/L)	25 (19–32)	27 (20–39)	0.303
γGT (U/L)	29 (23–52)	38 (29–54)	0.154
CK (U/L)	109 (71–158)	120 (92–162)	0.297
LDH (U/L)	175 (160–200)	174 (159–201)	0.818
Glucose (mmol/L)	**5.2 (5.1–5.7)**	**5.8 (5.2–6.7)**	**0.012**
Insulin (mU/L)	13.2 (9.3–16.6)	16.2 (9.9–31.2)	0.133
HbA1C (%)	**5.5 (5.2–5.9)**	**5.9 (5.5–6.5)**	**0.009**
Cholesterol (mmol/L)	5.08 ± 1.10	5.25 ± 1.01	0.506
LDL-C (mmol/L)	3.25 ± 0.96	3.21 ± 1.09	0.885
HDL-C (mmol/L)	**1.4 (1.2–1.6)**	**1.1 (0.90–1.2)**	**< 0.001**
Apo-A1 (g/L)	**1.58 ± 0.23**	**1.35 ± 0.20**	**< 0.001**
ApoB (g/L)	1.01 ± 0.25	1.11 ± 0.29	0.134
Lp(a) (mg/L)	**390.52 ± 455.97**	**150.51 ± 205.51**	**0.005**
TG (mmol/L)	**1.1 (0.7–1.6)**	**1.8 (1.5–3.6)**	**< 0.001**
CRP (normal/high)	**27/2**	**27/12**	**0.016**
Beck score	5 (2.5–10.5)	6 (3–10)	0.608
Epworth score	**8.21 ± 4.27**	**11.38 ± 4.48**	**0.004**
PSQI	4 (3–8)	5 (4–7.5)	0.169
Oxygen desaturation index (1/h)	**8 (6–16)**	**60.1 (29.9–84.4)**	**< 0.001**
Arousal index (1/h)	**26 (15.8–48.1)**	**48.1 (32.1–63.5)**	**0.002**
Central apnea index (1/h)	**1.1 (0.6–1.9)**	**3.3 (0.95–7.7)**	**0.002**
Obstructive apnea index (1/h)	**4.4 (3.0–7.0)**	**22.65 (15.65–41.1)**	**< 0.001**
Hypopnea index (1/h)	**7.8 (5.6–12)**	**19.8 (14.1–26.8)**	**< 0.001**

Patients were grouped into mild to moderate (AHI at < 30/h, *n* = 29 patients) or severe OSAHS (AHI ≥ 30/h, *n* = 40 patients). Data are presented as mean ± SD or median (interquartile range) unless otherwise stated. Differences between the two groups were considered significant at *p* < 0.05 (indicated in bold)

### Associations between Epworth sleepiness score, serum irisin and BDNF levels

Upon assessing the linear relationship between ESS and serum irisin levels, we found that their association is on the verge of statistical significance (*p* = 0.051), and no significant association was seen between ESS and serum BDNF levels (β = 0.008 (− 0.0023; 0.017, *p* = 0.129). If the influence of the interaction between serum irisin and BDNF were assessed, the results were yet again close to statistical significance (*p* = 0.055). Significant regressors of irisin included (log) weight, and (log) triglyceride, while serum BDNF showed a significant association with the number of people living in the patient’s household. ESS showed significant relationships with gender, number of people living in the same household, several polysomnography parameters, measures reflective of obesity, (sqrt) Beck depression score and (log) PSQI (Table 3).

**Table 3:** Simple linear regression model for the Epworth Sleepiness Scale and serum irisin and BDNF levels

Parameter	Coefficient (95% CI)	*p*
Simple linear regression of irisin		
Age in years	−0.016 (−0.035; 0.002)	0.083
Gender (f/m)	0.087 (−0.385; 0.560)	0.713
Epworth score	0.043 (−0.000; 0.086)	0.051
(log)weight	1.019 (0.032; 2.006)	**0.043**
(log)triglyceride	−0.317 (− 0.610; − 0.023)	**0.035**
Simple linear regression of BDNF		
Age in years	−1.986 (−4.525; 0.554)	0.123
Gender (f/m)	**−**40.476 (−103.752; 22.799)	0.206
No. in household	23.274 (0.020; 46.528)	0.050
Simple linear regression of Epworth score		
Age in years	−0.020 (−0.125; 0.086)	0.711
Gender (f/m)	2.91 (0.460; 5.370)	**0.021**
No. in household	0.981 (0.035; 1.927)	**0.042**
Irisin (ng/mL)	1.321 (−0.007; 2.649)	0.051
AHI (1/h)	0.083 (0.040; 0.125)	**< 0.001**
Oxygen desaturation index (1/h)	0.980 (0.050; 1.910)	**0.039**
(sqrt)Obstructive apnea index (1/h)	1.01 (0.49–1.55)	**< 0.001**
(log)PSQI	2.427 (0.812; 4.042)	**0.004**
(sqrt)Beck	1.323 (0.398; 2.249)	**0.006**
(log)weight	8.477 (3.309; 13.645)	**0.002**
1/BMI	−0.0252 (−0.045; 0.005)	**0.015**
Neck circumference (cm)	0.330 (0.128; 0.532)	**0.002**
(log)abdominal circumference	13.296 (5.168; 21.425)	**0.002**

Regression coefficient values are presented with their 95% confidence intervals (for the whole population of OSAHS patients: *n* = 69). The initial model for the multiple regression analysis consisted of the significant parameters provided by the simple regression and the relevant a priori identified parameters (statistical significance is indicated in bold)

**Fig. 1:**
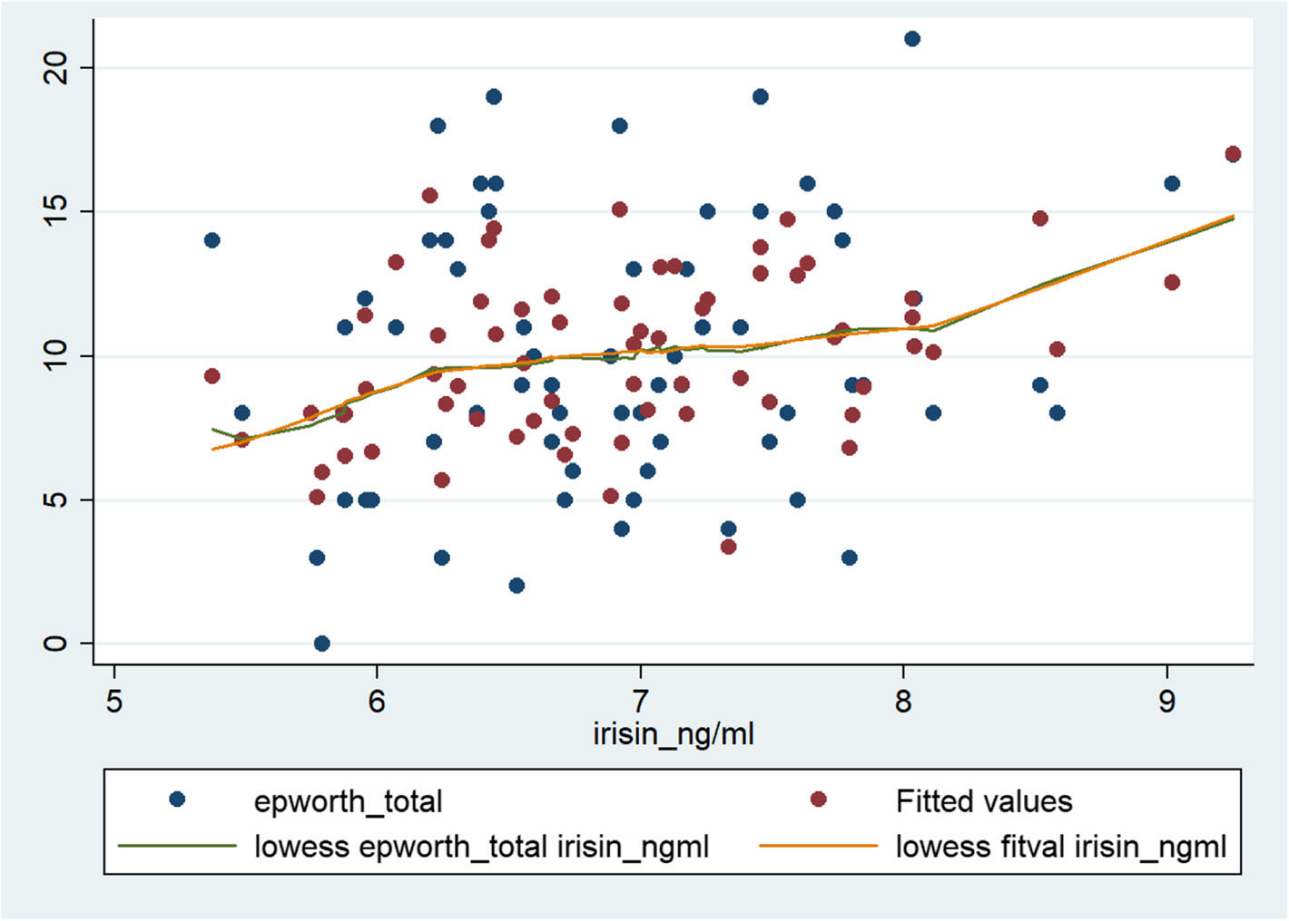
The model describing the linear relationship between Epworth Sleepiness Scale score and serum irisin and BDNF concentration in the whole population (*n* = 69). The x-axis shows the serum irisin concentration (in μmol/L), while the y-axis denotes the ESS score. The blue and red dots indicate the raw (i.e. original) values and fitted values obtained by multiple linear regression, respectively. The green and orange lines indicate the curves fitted to the raw data and to data provided by multiple linear regression. Fitting was done by locally weighted scatterplot smoothing (lowess)

**Fig. 2:**
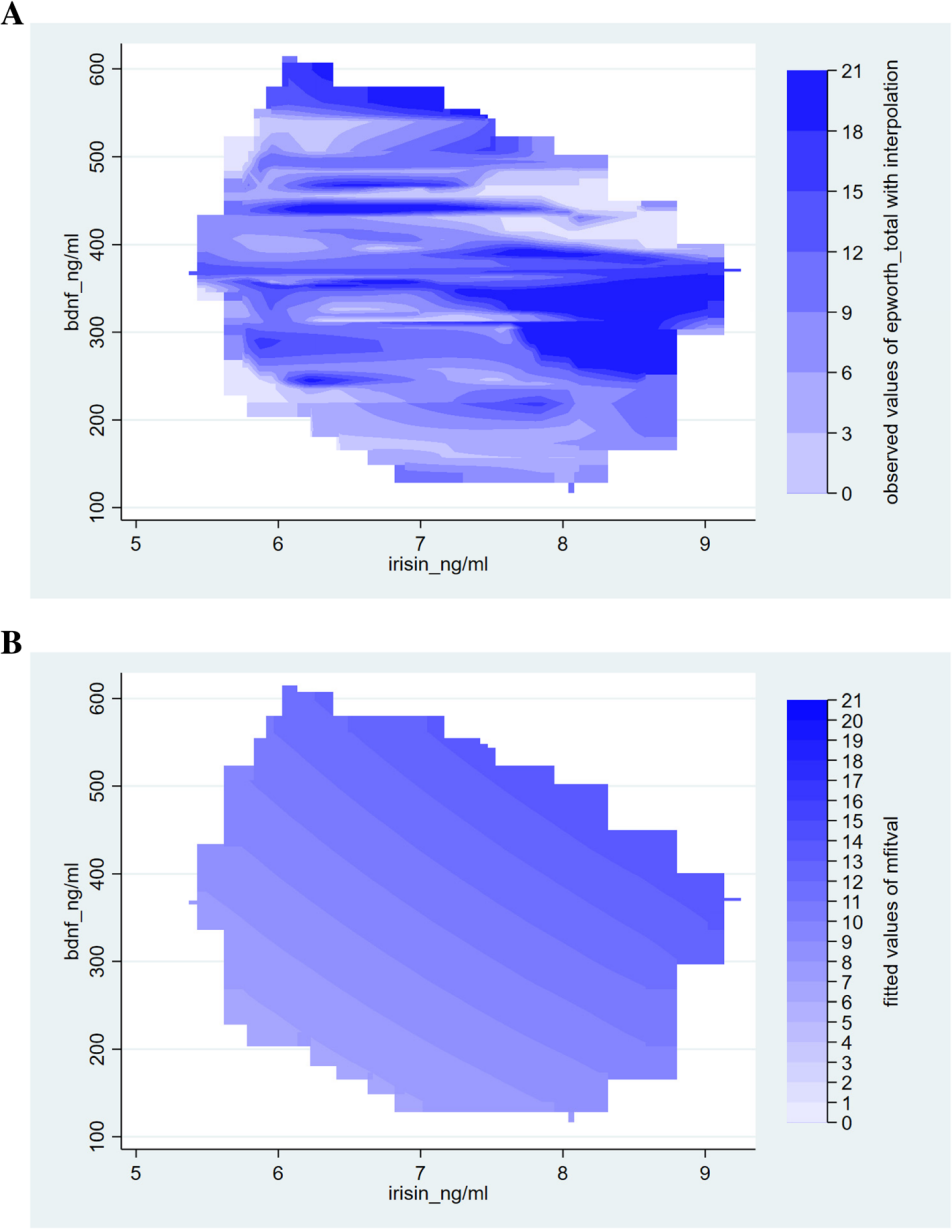
Association between Epworth Sleepiness Scale scores and serum irisin and BDNF levels. Panel A shows the observed values of ESS (axis z) as a function of serum irisin and BDNF using interpolation within data coverage limits, while Panel B is a contour map of fitted values of ESS as predicted by a multiple regression model with interaction between irisin and BDNF

**Fig. 3:**
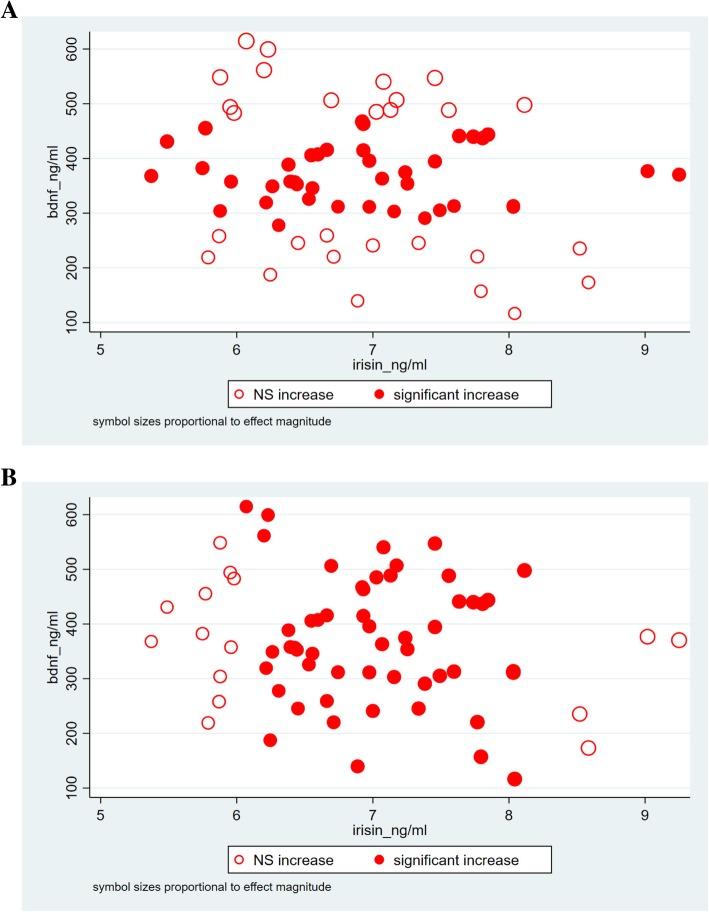
Scatter plots of serum BDNF versus serum irisin levels. Locations of model-estimated significant increase of ESS in response to a unit increase in serum irisin (Panel **a**) and serum BDNF (Panel **b**) are indicated by filled circles. Symbol sizes are proportional to effect magnitude

This relationship between ESS and the two independent variables of interest, irisin and BDNF showed strong significant association in the final multiple regression model (β_irisin_: 1.53; CI: 3.55, 2.70; *p* = 0.012; β_BDNF_: 0.014; CI: 0.005, 0.023; *p* = 0.002) that contained a priori determinants and (log) PSQI. Furthermore, significant interaction between serum irisin and BDNF levels (Table 4) were identified by the final model. The models not containing and containing the interaction between the two explanatory variables of interest were significant (*p* < 0.001 for both models). The Cook-Weisberg test showed no heteroskedasticity for either model (*p* = 0.08 and p = 0.08, respectively). The Ramsey test showed good fit for both models (*p* = 0.42; *p* = 0.46, respectively). Furthermore, good fit was also reflected by the locally weighted scatterplot smoothing (model devoid of the interaction term) (Fig. 1) and also by visual comparison of the three dimensional plots (axis x, y and z presenting serum irisin, BDNF levels and ESS, respectively) for the original data set and that fitted to the data yielded by our model (containing the interaction term) (Fig. 2). When plotting the final model addressing the interaction between irisin and BDNF, it is interesting to note that significant changes in the ESS are experienced if serum irisin level changed by 1 ng/mL, given the serum level of BDNF is within the range of ~ 280 to 470 ng/mL (Fig. 3a). Vice versa, ESS shows a significant increase in response to 1 ng/mL increase of BDNF if serum irisin levels are within the range of ~ 6.1 to 8.1 ng/mL (Fig. 3b). The final multiple linear regression model (built for the ESS) contained only (log) PSQI additional to the a priori identified parameters. The model showed that sleep quality is significantly associated with daytime drowsiness experienced by OSAHS patients (β: 7.01; CI: 0.85, 13.35; *p* = 0.026) (this relationship was significant whether or not the interaction term was included in the model).

**Table 4:** Multiple linear regression model for the Epworth Sleepiness Scale (ESS) and serum irisin and BDNF levels

Panel A	Coefficient (95% CI)	*p*
Age in years	0.02 (−0.091; 0.095)	0.970
Gender (f/m)	3.955 (1.761; 6.149)	**0.001**
Irisin (ng/mL)	1.530 (0.355; 2.705)	**0.012**
BDNF (ng/mL)	0.014 (0.005; 0.023)	**0.002**
(log) PSQI	2.972 (1.506; 4.437)	**< 0.001**
Panel B	Coefficient (95% CI)	*p*
Age in years	0.001 (−0.092; 0.095)	0.974
Gender (f/m)	3.992 (1.768; 6.216)	**0.001**
Irisin (ng/mL)	0.973 (−2.898; 4.844)	**0.006**
BDNF (ng/mL)	0.003 (−0.069; 0.075)	
Irisin-BDNF interaction term	0.002 (−0.009; 0.012)	
(log) PSQI	2.965 (1.487; 4.442)	**< 0.001**

Regression coefficient values are presented with their 95% confidence intervals (CI). The initial model for the multiple linear regression analysis consisted of the significant parameters provided by the simple linear regression and the relevant a priori identified parameters (age, gender) (statistical significance is indicated in bold)

## Discussion

The main finding of our study is that circadian misalignment indicated by excessive daytime sleepiness (characterized by the ESS) has a strong positive linear relationship with serum irisin and BDNF levels in a multiple linear regression model that corrects for age, gender and the only other significant regressor, (log) PSQI. Furthermore, we identified an interaction between serum irisin and BDNF levels with respect to their effect on ESS, suggestive of a concentration-response relationship. Our findings suggest that the effect of irisin on BDNF and subsequently on ESS follows the kinetics of the Hill equation [58]. Accordingly, in our OSAHS patient sample, increasing serum irisin level by 1 ng/mL fails to cause a significant increase of the ESS score if serum BDNF levels are in the lower range (e.g. below 280 ng/mL), indicating that low irisin levels have a small, nonsignificant effect on BDNF levels and consequently on ESS score. Conversely, changing serum irisin level by 1 ng/mL also fails to exert a significant increase in the outcome measure of interest at high serum BDNF levels (e.g. above 470 ng/mL), implicating that a theoretical maximal effect (E_max_) has been reached.

Similarly, increasing serum BDNF level by 1 ng/mL only leads to a significant increase in the ESS score if serum irisin levels are midrange e.g. fall between 6.1 ng/mL and 8.1 ng/mL.

These latter findings further support our theoretical presupposition that serum irisin is only one of several factors influencing BDNF levels, hence it may be altered independent of irisin (hence both parameters were included in the multiple model, as BDNF levels are subject to alter even at constant irisin levels).

Accordingly, BDNF expression is induced by metabolic changes accompanying the transition from carbohydrate and glucose utilization to fatty acids and ketones, a metabolic switch characteristic of intermittent fasting and exercise. Beta-hydroxybutyrate per se has been shown to induce BDNF expression with respect to metabolic switching, a finding reproduced in cultured cerebral cortical neurons under conditions of low glucose levels. Conversely, beta-hydroxybutyrate was shown to inhibit histone deacetylases, a repressor of BDNF expression [59]. Further potential sources of systemic BDNF level, independent of irisin’s effects includes platelets [60], and various tissues of the lung including the airway smooth muscle, bronchial epithelium, smooth muscle cells and peripheral nerve endings [61]. Hence, it is highly probable that the BDNF levels may vary due to processes that are independent of each other.

Worthy of notice is the finding that (log) PSQI was also found to be a significant predictor in the final multiple model especially if one considers the concept of two process model of sleep [22]. Our results suggest that poorer sleep quality (indicated by higher PSQI scores) results in more pronounced subjective daytime sleepiness. Allowing a more speculative interpretation, this model may intuitively posit that in the context of OSAHS alteration of sleep-related processes should be interpreted in terms of disrupted sleep homeostasis evolving due to intermittent airway obstruction, consequent hypoxia, and arousals and also via the alteration of the intertwining circadian regulatory processes reflected by the complex interaction between excessive daytime sleepiness and the irisin/BDNF axis. This interpretation provides a sound explanation for the phenomena of excessive daytime sleepiness reported in CPAP-treated OSAHS patients, in whom objective polysomnography-derived indices of OSAHS were shown to be normalized, while daytime sleepiness was preserved along with increased risk for morbidities and mortality [28, 29]. Nevertheless, recent advances in sleep research increasingly articulate that circadian and homeostatic processes are mutually influential, hence it is highly difficult to tease these inferences apart [22, 62].

The molecular effectors of photic entrainment in the SCN are via glutamate and pituitary adenylate cyclase-activating polypeptide (PACAP) [10, 21]. Contribution of BDNF, acting thru its cognate TrkB receptor to this process has been described previously [21]. Dedicated retinohypothalamic fibers containing blue light sensitive photopigments terminate in the SCN where pre- and postsynaptic TrkB receptors are expressed near BDNF-expressing SCN cells. Hence the spatial relationship permits modulating the synaptic interaction [21, 63]. Conversely, BDNF enhances the release of glutamate and PACAP [21, 63, 64] and augments postsynaptic response to glutamate via several mechanisms. It phosphorylates NMDA receptors, increasing the opening probability of the channel in mice and rat [63, 65] and/or increases the number of cell surface NMDA receptors by inducing rapid cycling of the receptor [63].

The expression of BDNF within the SCN shows a rhythmic pattern, elevating during subjective night from the basal daytime level [66].During the day, transmission of the light signal through the retinohypothalamic tract-SCN synapse is absent given the insufficiency of basal BDNF level to permit excitatory neurotransmitter release [21]. Accordingly, photic stimuli cannot entrain the master clock, rendering the SCN insensitive to perturbations of light during the subjective day, while at night the SCN responds to light by markedly shifting the phase of this clock [21]. The fact that BDNF administered into the SCN of rats permits phase shifts in response to light during subjective day further corroborates BDNF’s gating function [67, 68]. Moreover, photic entrainment is diminished in BDNF-deficient knockout mice, and in rats if the tyrosine kinase inhibitor K252a is infused into their SCN [21, 67]. Hence BDNF has a permissive effect in the SCN gating light’s ability to entrain the master circadian clock in the SCN.

Prior work has suggested that BNDF is mandatory for glutamate to induce the signal-transduction cascade that mediates the light induced shift and thus photic entrainment of the circadian system ensuring that the organism’s internal circadian rhythm is aligned with the geophysical day. Thus, it may be concluded that BDNF assumes a critical role in photic entrainment of the master clock by gating the circadian system to light [21, 63], and irisin may modulate this effect by influencing BDNF levels in the hypothalamus.

A few clinical studies are available concerning the change of neurotrophins in OSAHS. One early study evaluated the expression profile of BDNF in adenotonsillar tissue obtained from children undergoing adenotonsillectomy. Children were either suffering from OSAHS or recurrent tonsillitis. No significant difference in the mRNA levels was found between these two patient groups [69]. A clinical trial including middle-aged OSAHS patients (15 female and 24 male patients) and controls (24 men) reported no significant difference in average serum BDNF levels (11.22 ± 0.46 ng/mL vs. 11.17 ± 0.41 ng/mL for patients and controls, respectively) [70]. The serum levels reported in the present investigation are considerably higher, but fall within the range reported by others. Studies including healthy individuals and other patient populations reported serum BDNF levels spanning over four orders of magnitude, ranging from 0.005 ng/ml to 280 ng/ml, [71–74] using different ELISA kits. Our group has formerly reported serum BDNF levels of 345.6 ng/mL (IQR 294.20–387.90 ng/mL) and 314.46 ± 118.68 ng/mL in cohorts of chronic obstructive pulmonary disease patients [75] and bronchial asthma [76], respectively. To the best of our knowledge there are no reports concerning the serum level of irisin in OSHAS patients. In one study evaluating the effects of CPAP therapy in OSHAS patients, irisin levels were determined, however no absolute serum levels were reported, only changes from baseline values, mean difference between groups and correlation coefficients were published [77]. Previously our group has reported serum irisin levels in the range of 7.22 ng/mL (IQR: 6.63–8.10 ng/mL) in COPD and 7.87 ng/mL (IQR: 7.15–8.82 ng/mL) in asthma patients. Others have reported irisin levels of 26.3 (IQR: 22.6–32.4) ng/ml, 53.7 (IQR: 46.7–62.8) ng/ml, 58.5 (42.8–78.9) ng/ml in smokers with and without COPD, and in non-smoking individuals, respectively [78].

Choosing only the Epworth Sleepiness Scale to characterize the possible alteration of circadian rhythm could be regarded as a limitation of the current study. The ESS is considered as a standard questionnaire appropriate in the clinical setting to provide a subjective measure of daytime sleepiness [41]. Furthermore, that objective measure of excessive daytime sleepiness was not obtained should also be included among the limitations. It is acknowledged that daytime sleepiness may be caused by several diseases and conditions (e.g. poor sleep hygiene, primary hypersomnias, depression, obesity etc). These possible confounders were handled by recruiting only OSAHS patients (note that one of the diagnostic criteria for the clinical diagnosis of OSAHS is excessive daytime sleepiness that is not due to any other known causes e.g. primary hypersomnia), by excluding patients with certain co-morbidities (malignancies, kidney disease, COPD and asthma) and by controlling for parameters known to reflect conditions in which excessive daytime sleepiness may be present (e.g. BMI, neck circumference, Beck depression inventory score, included in the initial model). In OSAHS, excessive daytime sleepiness is generally considered to be the consequence of intermittent nocturnal hypoxemia that leads to sleep fragmentation, hence poorer sleep quality. To attest for the possible influence of reversible upper airway obstruction on daytime hypersomnolence, significant polysomnography derived indices (AHI, oxygen desaturation index and obstructive apnea index) were also included in the model as well as (log) PSQI gauging perceived nocturnal sleep quality. Previous studies have shown a poor association between ESS and PSQI as well as between PSQI, ESS and polysomnography measures [79] to the extent that it was suggested that these measures reflect distinct aspects of sleep. After controlling for causes other than altered circadian rhythm, we feel that ESS may be considered as an indicative parameter reflective of altered circadian rhythm in our explorative study. Nevertheless, characterization of the circadian rhythm by obtaining serial measurements of either salivary or serum melatonin or core body temperature could have added value at the cost of extra inconvenience for the patients and possibly have interfered with other measures e.g. polysomnography. However, alteration of the circadian rhythm in OSAHS has been established previously [37], allowing the speculative notion that altered irisin/BDNF axis may be causative for circadian misalignment in OSAHS, and thus, it may be suggested that excessive daytime sleepiness is a result of the deterioration of circadian pacemaker process. This alternative mechanism could account for the phenomenon of residual EDS as well as the excess risk of cardiovascular and all-cause morbidity and mortality in CPAP treated OSAHS patients. Measurements from a single timepoint may be considered as further limitation of the study, as serum BDNF levels are themselves circadian [80]. Nevertheless, the current findings suggest a putative relationship between the subjective measure of excessive daytime sleepiness and the alteration of the irisin/BDNF axis as reflected by the single measurement. Furthermore, this finding may contribute to teasing apart two parallel mechanisms underlying excessive daytime sleepiness e.g. one stemming from the mechanical obstruction of the airways and the other from the alteration of the circadian regulation.

Nevertheless, the present work has several merits. Presence of OSAHS was verified using overnight polysomnography conducted in an accredited sleep centre. Stringent data analysis should also be considered a further strength, as well as the fact that to the best of our knowledge, this is the first-time absolute values of serum irisin level are disclosed, and relatively little information is available regarding serum BDNF levels in OSAHS.

## Conclusion

The current work proposes that the sleep related disturbances, sleep quality and excessive daytime sleepiness, may be the outcome of distinct pathologies, e.g. mechanical obstruction, and deterioration of the circadian rhythm, respectively. Furthermore, based on the finding that excessive daytime sleepiness shows a strong association with the alteration of the irisin-BDNF axis, we put forward the speculative notion that these factors may be causative in the evolution of circadian misalignment, given the known involvement of BDNF in photic entrainment in the master clock of the SCN. Furthermore, our results indirectly support the putative role of irisin in altering the levels of BDNF at sites relevant regarding circadian regulation.

## Abbreviations

ACE: Angiotensin converting enzyme
AHI: Apnea-hypopnea index
apoA1: Apolipoprotein A1
apoB: Apolipoprotein B
ARB: Angiotensin receptor (type 1) blocker
BDNF: Brain-derived neurotrophic factor
BMI: Body mass index
CAD: Coronary artery disease
CI: 95% confidence interval
CK: Creatine kinase
COPD: Chronic obstructive pulmonary disease
CPAP: Continuous positive airway pressure
CRP: C-reactive protein
CVD: Cardiovascular disease
ELISA: Enzyme-linked immunosorbent assay
ESS: Epworth Sleepiness Scale
FNDC5: Fibronectin type III domain-containing protein 5
GOT: Glutamate-oxaloacetate transaminase
GPT: Glutamate-pyruvate transaminase
HbA1c: Hemoglobin A1c
HDL: High-density lipoprotein
IMT: Intima-media thickness (of common carotid artery)
IQR: Interquartile range
LDH: Lactate dehydrogenase
LDL: Low-density lipoprotein
Lp(a): Lipoprotein (a)
NMDA: N-methyl-D-aspartate
OSAHS: Obstructive sleep apnea hypopnea syndrome
PACAP: Pituitary adenylate cyclase-activating polypeptide
PSQI: Pittsburgh sleep quality index
RR: Blood pressure measured according to Riva-Rocci (modified by Korotkov)
SCN: Suprachiasmatic nucleus
TG: Triglyceride
TrkB: Tropomyosin receptor kinase B (tyrosine receptor kinase B)
γGT: Gamma-glutamyl transferase
